# Tick Ectoparasites of Animals in Borderline of Iran-Iraq and Their Role on Disease Transmission

**Published:** 2018-09-30

**Authors:** Omid Banafshi, Ahmad Ali Hanafi-Bojd, Mohamad Karimi, Faezeh Faghihi, Mojtaba Beik-Mohammadi, Sahere Gholami, Siavash Javaherizadeh, Hamideh Edalat, Hassan Vatandoost, Zakkyeh Telmadarraiy

**Affiliations:** 1 Zoonoses Research Center, Kurdistan University of Medical Sciences, Sanandaj, Iran; 2 Department of Medical Entomology and Vector Control, School of Public Health, Tehran University of Medical Sciences, Tehran, Iran; 3 Cellular and Molecular Research Center, Iran University of Medical Sciences, Tehran, Iran; 4 Department of Medical Entomology and Vector Control, Faculty of Health, Urmia University of Medical Sciences, Urmia, Iran; 5 Facalty of Paramedical Science, Clinical Laboratory Science, Islamic Azad University, Tehran Medical Branch, Tehran, Iran

**Keywords:** Tick, Ruminants, Turtle, Iran

## Abstract

**Background:**

Since ticks are potent vectors of various diseases, identification of these species are clinically important to protect the public health and control veterinary problems in the communities. We aimed to figure out the frequency of ticks on cows, goats, sheep, lambs, turtles and also obscure hosts in Kurdistan Province, bordered with Iraq June 2012 to May 2013.

**Methods:**

The hosts were selected randomly and examined individually for tick infestation. In case of infestation, ticks were collected using forceps and then preserved in 70% ethyl alcohol. All collected specimens were preserved in tubes and relative information was recorded and then identified based on morphological characteristics.

**Results:**

Totally, 1209 ticks were collected. The prevalence of ticks on cows, sheep, goats, lambs, turtles, poultry and obscure hosts was 11.33%, 55.41%, 6.53%, 5.95%, 0.9%, 8.02% and 11.82% respectively. The mean number of ticks on each animal was 1.6. Number of 5 genera, including *Rhipicephalus*, *Argas*, *Ornithodoros*, *Hyalomma* and *Haemaphysalis* and 9 species; including *R. sanguineus* (60.05%), *R. bursa* (0.08), *Hy. anatolicum* (12.33), *Hy. asiaticum* (1.49), *Hy. aegyptium* (0.91), *Hy. marginatum* (0.08), *Haemaphysalis parva* (4.22), *Hyalomma* sp. (0.99), *Ornithodoros lahorensis* (11.83), and *Argas persicus* (8.02) were identified.

**Conclusion:**

The most abundant species in this study area was *Rh. sanguineus* (60.05%). Due to high prevalence of tick specimens and a variety of collected species from sheep (55.41%), the vaccination of sheep and control of tick vectors are recommended.

## Introduction

Ticks (Acari: Ixodidae) are considered as the important vectors of pathogens (1). They play an important role in the survival of the pathogens that cause disease in humans and animals (2). Ticks are able to transmit a variety of pathogens that are responsible to develop some diseases such as tick-borne encephalitis, Crimean Congo Hemorrhagic Fever (CCHF), anaplasmosis, babesiosis, rick-ettsiosis, borreliosis and ehrlichiosis (3, 4). Such diseases are considered as public health or veterinary problems in the countries (5, 6). To the best of our knowledge, 10% of the currently known tick species act as vectors of pathogens of animals and humans (2). In addition to the transmission of pathogens, they are also responsible for damages directly due to their feeding behavior (7). As only 10% of tick species transmit a number of pathogens, identification of tick species is important. Tick species distribution in Iran is briefly investigated on the basis of published records. data were presented for 642 ixodid ticks taken from small-sized mammals, mainly rodents in different zoogeographical zones of Iran (8). The prevalence of ixodid ticks was studied on cattle in Mazandaran Province, north of Iran (9) and east of the country (10) in another study the prevalence of ticks was investigated in Khuzestan Province and showed Shosh was the most infected city in Khuzestan, Ticks infection rate on sheep, goat, and cow was 84.12%, 12.69% and 3.17%, respectively (11).

In a similar investigation, the distribution and ecological preferences of ticks of domestic animals were studied from 2002 to 2005 in north part of the country (12). The prevalence of ticks was surveyed in north-west of the country in Ardebil (13) and West Azerbaijan (14). Additionally, hard ticks of domestic ruminants were surveyed in central part of Iran (15). Recently, some other investigations have been carried out in some other geographical locations of Iran (16–19), and in Kurdistan region of Iran and Iraq (20–22). In 2002, the presence of *Hy. aegyptium* from *Testudo graeca* turtle was reported in Iran (23). *Hy. aegyptium* and *T. graeca* were found in northwest of Iran (24). Recently the situation of tick born disease showed in Iran. The CCHFV RNA was detected in 5.2% of 492 ticks collected from livestock in different regions of Golpayegan (6). In total, 49 ticks including five species: *R. sanguineus*, *Hyalomma anatolicum*, *Hy. asiaticum*, *Hy. dromedarii* and *Hy. marginatum* with a prevalence of 46.9%, 32.7%, 4.1%, 4.1% and 2.1% respectively were identified; and CCHFV was detected in three ticks among 49 collected ticks. The ticks infected with CCHFV belonged to the genus *Hyalomma* and *Rhipicephalus*. Phylogenetic analysis demonstrated that two sequences clustered in clade IV (Asia-1) and one sequence was located within clade IV (Asia-2) (25). All positive ticks were from *Hyalomma* genus and *Hy. marginatum* species. They were not able to find virus in *Hy. anatolicum*, *Hy. schulzei*, *Hy. dromedarii*, *R. sanguineus* and *Argas persicus. Hyalomma marginatum* is the main vector in that study (26)

Despite the aforementioned investigations, there still seems to be a gap in our knowledge about distribution of tick species in Iran. This study was aimed to figure out the frequency of ticks on cows, goats, sheep, lambs, turtles and also obscure hosts in Kurdistan Province, bordered with Iraq.

## Materials and Methods

**Fig. 1.**
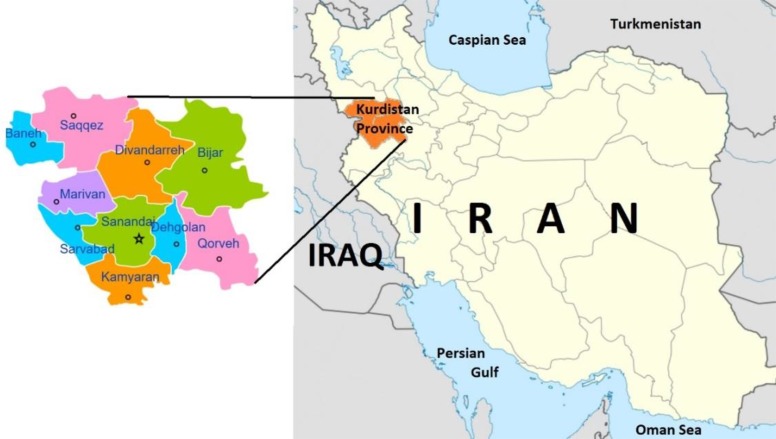
The study area, Kurdistan Province is located in west part of Iran

This survey was carried out in Kurdistan Province, located in west part of Iran, in Region 3 and bound by Iraq on the west, the province of West Azerbaijan to its north, Zanjan to the northeast, Hamedan to the east and Kermanshah to the south (27). This province is one of the 31 provinces of Iran. It is 28817km^2^ in area (Coordinates: 35.3113°N 46.9960°E). The capital of Kurdistan Province is the city of Sanandaj, located in Sanandaj County. Other counties with their major cities are Marivan, Baneh, Saqqez, Qorveh, Bijar, Kamyaran, Dehgolan, Diwandarreh and Sarvabad (Fig. 1).

### Samples collection

From June 2012 to May 2013, ticks from goats, cows, sheep, lambs, turtles and obscure hosts from various regions of the province were collected. Ticks were mostly found on sheep of the livestock. In total, 724 animals from 104 herds including 62 cows, 506 sheep, 73 goats and 23 lambs were selected randomly and examined individually for tick infestation. Additionally, we selected 2 turtles and 37 obscure hosts randomly for detection of tick infestation on them. Thirty minutes were spent for each flock to collect ticks. All inspections and tick collections were carried out between 08:00 a.m. and 11:00 a.m. In case of infestation, ticks were collected using forceps and then preserved in 70% ethyl alcohol. Collected samples were preserved in tubes and relative information was recorded such as collector name, date, host information and date of collection, then, samples were transferred to the Entomology Laboratory, School of Public Health, Tehran University of Medical Sciences, Tehran, Iran. All collected samples were identified based on morphological characteristics and the keys given by Janbakhsh (28) and Walker et al. (29) based on shape of capitulum, scutum, eyes, festooned and hypostome, spiracle, genital groove, spure of coxa, adanal shield and other appropriate characters.

## Results

**Fig. 2.**
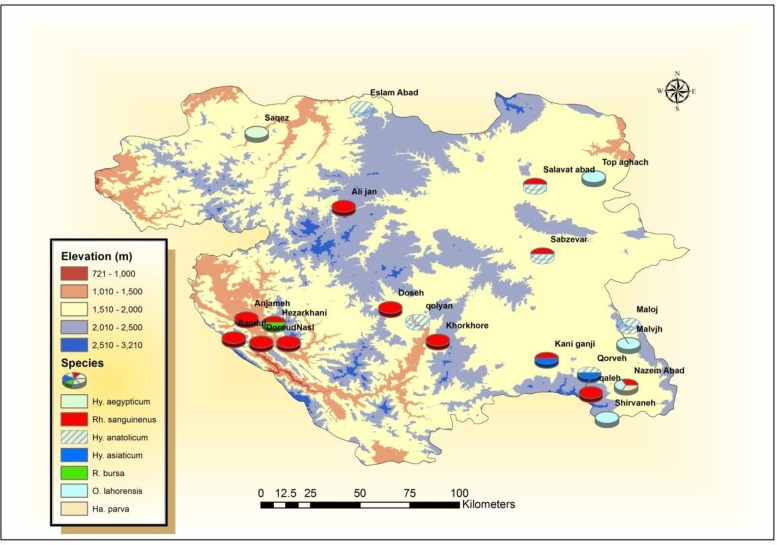
Spatial distribution of livestock ticks in different altitudinal categories of Kurdistan Province of Iran, 2012–2013

Totally 1209 ticks were collected and the occurrence of ticks on cows, sheep, goats, lambs, turtles, poultry and obscure hosts was 11.33%, 55.41%, 6.53%, 5.95%, 0.9%, 8.02% and 11.82% respectively. The mean number of ticks on each animal was 1.6 ticks per animal. Totally 5 Genus: *Rhipicephalus*, *Argas*, *Ornithodoros*, *Hyalomma* and *Haemaphysalis* were identified in study areas (Table 1). *Rhipicephalus sanguineus* was the most abundant species in the studied area (60.05%), also, we found *R. bursa* (0.08%), *Argas persicus* (8.02 %), *Ornithodoros lahorensis* (11.83%), *Hy. marginatum* (0.08%), *Hy. asiaticum* (1.49%), *Hy. anatolicum* (12.33%), *Hy. aegyptium* (0.91 %), *Ha. parva* (4.22%) and *Hyalomma* sp. (0.99%). Spatial distribution of tick species in different elevations is presented in Fig. 2.

**Table 1.** Tick species and their hosts and places in Kurdistan Province, 2012–2013 (N: Nymphs, F: Females, M: Males)

**Species of ticks**	**Host and Place (%)**							**Collected samples**			**Total (%)**

	**Cow**	**Sheep**	**Goat**	**Lamb**	**Turtle**	**Poultry**	**Fold**	**N**	**F**	**M**	
***R. sanguineus***	17	572	65	72	0	0	0	53	200	473	726 (60.05)
***R. bursa***	0	1	0	0	0	0	0	0	0	1	1 (0.08)
***Hy. anatolicum***	114	35	0	0	0	0	0	0	88	61	149 (12.33)
***Hy. asiaticum***	0	5	13	0	0	0	0	0	6	12	18 (1.49)
***Hy. aegyptium***	0	0	0	0	11	0	0	0	9	2	11 (0.91)
***Hy. marginatum***	0	0	1	0	0	0	0	0	0	1	1 (0.08)
***H. parva***	0	51	0	0	0	0	0	0	20	31	51 (4.22)
***Hy.* sp**	6	6	0	0	0	0	0	1	11	0	12 (0.99)
***O. lahorensis***	0	0	0	0	0	0	143	122	8	13	143 (11.83)
***A. persicus***	0	0	0	0	0	97	0	63	21	13	97 (8.02)
**Total**	137 (11.33)	670 (55.41)	79 (6.53)	72 (5.95)	11 (0.90)	97 (8.02)	143 (11.82)	239 (19.77)	363 (30.02)	607 (50.21)	1209 (100)

The coordinates of collection sites have presented in Table 2.

**Table 2.** Spatial distribution of ticks collected from the study area, Kurdistan Province of Iran, 2012–2013 (+: Collected,−: Not collected)

**County**	**Village**	**X**	**Y**	**Host**	***Hy. aegyptium***	***Rh. sanguineus***	***Hy. anatolicum***	***Hy.asiaticum***	***R.bursa***	***O. lahorensis***	***Ha.parva***
**Bijar**	Khorkhore	47.1094	35.3086	Sheep	−	+	−	−	−	−	−
**Bijar**	Sabzevar	47.5833	35.7	Sheep	−	+	+	−	−	−	−
**Bijar**	Bijar-salavatabad	47.55	36.0166	Sheep	−	+	−	−	−	−	−
**Bijar**	Salavatabad	47.55	36.0166	Cow	−	−	+	−	−	−	−
**Bijar**	Salavatabad	47.55	36.0166	Cow	−	−	+	−	−	−	−
**Bijar**	Salavatabad	47.55	36.0166	Goat	−	+	−	−	−	−	−
**Bijar**	Top aghach	47.8161	36.0508	Sheep	−	−	−	−	−	+	−
**Bijar**	Top aghach	47.8161	36.0508	Sheep	−	−	−	−	−	+	−
**Qorveh**	qaleh	47.4810	35.0803	sheep	−	+	−	−	−	−	−
**Qorveh**	Maloj	47.9733	35.2922	sheep	−	−	+	−	−	−	−
**Qorveh**	Kaniganji	47.3611	35.1332	Sheep	−	−	−	+	−	−	−
**Qorveh**	Kaniganji	47.3611	35.1332	Sheep	−	+	−	−	−	−	−
**Qorveh**	Qorveh	47.7951	35.1594	Goat	−	−	+	+	−	−	−
**Qorveh**	Qorveh	47.7951	35.1594	Sheep	−	−	−	−	−	−	−
**Qorveh**	Malvjh	47.9733	35.2922	Goat	−	−	−	−	−	+	−
**Qorveh**	Nazem Abad	47.9625	35.1038	Sheep -Goat	−	−	−	−	−	−	+
**Qorveh**	Nazem Abad	47.9625	35.1038	Sheep -Goat	−	−	−	−	−	+	−
**Qorveh**	Nazem Abad	47.9625	35.1038	Sheep -Goat	−	+	−	−	−	−	−
**Qorveh**	Shirvaneh	47.8755	34.9566	Goat	−	−	−	−	−	+	−
**Sanandaj**	Ali jan	46.6833	35.9166	Sheep	−	+	−	−	−	−	−
**Sanandaj**	Doseh	46.8955	35.4566	sheep	−	+	−	−	−	−	−
**Sanandaj**	qolyan	47.0106	35.2355	sheep	−	−	+	−	−	−	−
**Saqez**	Saqez	46.2892	36.2523	Turtle	+	−	−	−	−	−	−
**Saqez**	Eslam Abad	46.7666	36.3666	Sheep	−	−	+	−	−	−	−
**Sarvabad**	Anjameh	46.35	35.3666	Goat	−	+	−	−	−	−	−
**Sarvabad**	Anjameh	46.35	35.3666	Cow	−	+	−	−	−	−	−
**Sarvabad**	Bandul	46.1535	35.1911	Sheep	−	+	−	−	−	−	−
**Sarvabad**	Doroud	46.3533	35.2994	Sheep/♀	−	+	−	−	−	−	−
**Sarvabad**	Doroud	46.3533	35.2994	lamb/♀	−	+	−	−	−	−	−
**Sarvabad**	Hezarkhani	46.3666	35.35	Sheep/♀	−	+	−	−	+	−	−
**Sarvabad**	Hezarkhani	46.3666	35.35	Sheep/♀	−	+	−	−	−	−	−
**Sarvabad**	Hezarkhani	46.3666	35.35	Goat /♀	−	+	−	−	−	−	−
**Sarvabad**	Hezarkhani	46.3666	35.35	lamb/♀	−	+	−	−	−	−	−
**Sarvabad**	Nasl	46.4333	35.3	Sheep/♀	−	+	−	−	−	−	−
**Sarvabad**	Nasl	46.4333	35.3	Sheep/♀	−	+	−	−	−	−	−
**Sarvabad**	Nasl	46.4333	35.3	sheep/♂	−	+	−	−	−	−	−
**Sarvabad**	Nasl	46.4333	35.3	Cow/♀	−	+	−	−	−	−	−
**Sarvabad**	Nasl	46.4333	35.3	Sheep	−	+	−	−	−	−	−
**Sarvabad**	Nasl	46.4333	35.3	Sheep/♀	−	+	−	−	−	−	−

## Discussion

Ticks are considered as ectoparasites, living by hematophagy on the blood of mammals, birds, and sometimes reptiles and amphibians. About 10% of Ixodidae (hard ticks) and Argasidae (soft ticks) are vectors of a number of diseases that affect both humans and other animals. As ticks are important vectors of diseases; they are subject of many studies in Iran. Due to former investigations, there is limited information about distribution of tick infestation in Kurdistan Province.

In most regions of Iran, the dominant tick genera responsible for infestation belong to *Hyalomma*, *Rhipicephalus*, *Haemaphysalis*, and *Ixodes* (30). In this investigation, we could collect three of the aforementioned genera except for *Ixodes* but also we collected some species of genera *Argas* and *Ornithodoros* too.

We collected 1209 ticks. Most of the collected ticks were male (50.21%) (Table 1). Our survey revealed that the most occurrences of ticks were observed on sheep (55.41%). The identification of collected ticks also revealed that the occurrence of ticks on cows, goats, lambs, turtles and obscure hosts were 11.33%, 6.53%, 5.95%, 0.9% and 11.82% respectively. As haemoparasitic diseases are considered as a major problem to efficient sheep production in Iran due to theileriosis and babesiosis, it has important role (30). The major tick genera found on sheep and goats are mostly *Hyalomma*, *Rhipicephalus*, *Haemaphysalis* and *Ixodes* (30). Our investigation revealed the presence of all species of ticks except *Hy. marginatum* and *Hy. aegyptium* on sheep. Due to high prevalence of tick specimens and variety of collected species of sheep, the vaccination of sheep and control of tick vectors are recommended.

An investigation in Kurdistan region in Iraq was carried out (22). Three genera species were collected and identified on cattle. The highest prevalence was observed in *Boophilus* sp. followed by *Hyalomma* sp. and *Rhipiceph alus* sp. (22). We could not detect any *Boophilus* species, but we found *R. sanguineus* and *Hy. anatolicum* on cows. These findings are in concordance with the mentioned investigation (22).

Our investigation shows the presence of 9 species: *R. sanguineus* (60.05%), *R. bursa* (0.08), *Argas persicus* (8.02), *Ornithodoros lahorensis* (11.83), *Hy. marginatum* (0.08), *Hy. asiaticum* (1.49), *Hy. anatolicum* (12.33), *Hy. aegyptium* (0.91), *Ha. parva* (4.22) and *Hyalomma* sp. (0.99) in the province. *R. sanguineus* was the most collected tick sample (726/1209), also, these species were found on all hosts except turtles (Table 1). *Rh. sanguineus* (brown dog tick) is considered as the most widespread ixodid tick, colonizing both human and animals (31). *R. Sanguineus* species are very resistant to heat and moisture deficits (32). This species is able to transmit pathogens like *Ehrlichia canis* to dog (33). They can participate in the epidemiology of canine visceral leishmaniasis (34) and spotted fever group rickettsia (35). Some other Ehrlichia associated species in *R. sanguineus* are *E. ewingii*, and *E. chaffeensis* (36).

The brown dog tick is also able to transmit *Rickettsia ricksettsii*, causing Rocky Mountain Spotted Fever (37), *Rickettsia conorii*, which is the bacteria responsible for causing Mediterranean spotted fever as well as *Rickettsia massiliae* (38) and *R. massiliae* (39). *Rhipicephalus sanguineus* is also reported to transmit *Hepatozoon canis* (40) as well as *Babesiacanis* (41).

## Conclusion

Ticks contamination has been detected in a variety of livestock in the Kurdistan region, also the variety of ticks is abundant. The *Ornithodoros* and *Hyalomma* are more important than carriers of known diseases in the Kurdistan, including Crimean Congo hemorrhagic fever (CCHF) and tick-borne relapsing fever (TBRF).

Regarding the high contamination of livestock, the presence of disease and borderline province, the importance of the vector control in Kurdistan is more evident, as well as the necessity of further research, especially on the movement of livestock and ticks, as well as the resistance of ticks to the pesticide.
